# Anti-infective potential, chemical profile, and molecular docking investigation on antioxidant-rich fraction of *Murraya koenigii* against Gram-negative pathogenic bacteria

**DOI:** 10.3389/fmicb.2026.1739591

**Published:** 2026-02-04

**Authors:** Zarrin Haris, Iqbal Ahmad, Nayla Munawar, Mohd Adil, Maher Alandiyjany, Fohad Mabood Husain

**Affiliations:** 1 Department of Agricultural Microbiology, Faculty of Agricultural Sciences, Aligarh Muslim University, Aligarh, India; 2 Department of Chemistry, College of Science, United Arabs Emirates University, Al Ain, United Arab Emirates; 3 Department of Plant, Food and Environmental Sciences, Dalhousie University, Truro, NS, Canada; 4 Department of Clinical Laboratory Sciences, Faculty of Applied Medical Sciences, Umm Al-Qura University, Makkah, Saudi Arabia; 5 Department of Food Science and Nutrition, College of Food and Agriculture Sciences, King Saud University, Riyadh, Saudi Arabia

**Keywords:** biofilm inhibition, molecular docking, *Murraya koenigii*, phytochemistry, quorum sensing, virulence factors

## Abstract

**Purpose:**

To assess the antioxidant-rich fraction of *Murraya koenigii* for its anti-infective properties against Gram-negative pathogenic bacteria by *in vitro* and *in silico* approaches.

**Results:**

The most antioxidant active fraction, i.e., *M. koenigii* chloroform fraction (MKCF), significantly reduced violacein production (70.73%) in *Chromobacterium violaceum* 12,472. Significant reduction in prodigiosin production, protease activity, and swarming motility of *Serratia marcescens*, and other tested virulence factors of *Pseudomonas aeruginosa* PAO1 was recorded. More than 60% reduction in biofilm formation was recorded against test pathogens, indicating broad-spectrum anti-infective activity. SEM and CLSM imaging revealed alterations in the structure of the biofilm. Major key compounds such as Gibberellic acid, methyl ester, 7,8-Epoxylanostan-11-ol, 3-acetoxy were detected by GC/MS, and numerous compounds in MKCF were identified using LC-qTOF/MS analysis. *In silico* analysis revealed morellin and murrayazolinol with good binding affinity with CviR and EsaI, with binding energies of −9.07 and −9.17 kcal mol^−1^, respectively.

**Conclusion:**

The most active antioxidant fraction, i.e., MKCF, could be exploited as an anti-infective agent against Gram-negative bacterial pathogens, attenuating virulence and pathogenicity. Further, *in vivo* efficacy of the active fraction/phytocompounds needs to be evaluated to explore the therapeutic potential of MKCF.

## 1 Introduction

Treating bacterial infections has become extremely difficult due to the ongoing emergence and spread of multidrug-resistant bacterial pathogens. Addressing this issue requires the development of alternative and innovative strategies to combat microbial infections. Due to the adverse effects on the host’s microbiome and the resulting resistance, antibiotics can no longer be relied upon for long-term treatment (Maisuria et al., 2016). Quorum sensing (QS) is a cell-to-cell communication mechanism in bacteria that uses chemical signaling to provide density-dependent responses. In many pathogenic bacteria, QS is involved in pathogenesis and biofilm formation (Rutherford and Bassler, 2012). QS regulates the formation of biofilm in many bacteria. Bacterial biofilms are complex microbial communities encased in extracellular polymeric substances (Zhao et al., 2023). It is thought to be a crucial trait that increases the virulence of pathogenic bacteria. It is a dense collection of bacteria that has protected growth, which allows them to survive in harsh conditions, such as those found in human hosts. Biofilm formation has reportedly been linked to more than 80% of infections caused by pathogenic bacteria. Therefore, biofilms may be regarded as a special mode of persistent bacterial infection. In the biofilm mode of growth, microorganisms exhibit numerous drug resistance mechanisms like heterogeneity in metabolism and growth rate, increased expression of efflux pumps, drug hydrolysis by matrix components, the existence of persister cells and most importantly, a cell density-dependent communication phenomenon or quorum sensing (Hajiagha and Kafil, 2023; Singh et al., 2021).

As a result, further strategies that target bacterial QS are being investigated to attenuate virulence and pathogenicity. It is expected that inhibiting virulence rather than growth may reduce the likelihood of resistance developing.

Scientists are thus motivated to analyze and test the value of plant-derived compounds, leveraging their conventional applications in healthcare. Herbal medicine/plant-derived products are rich in diverse phytocompounds with multiple biological activities, including commonly encountered antioxidant activity (Dragland et al., 2003; Alok et al., 2014). Antioxidants are micronutrients that have become more significant in recent years because of their capacity to inhibit the activity of free radicals and reactive oxygen species. They mitigate or stop oxidative damage to a target molecule in organisms. It is believed that plant-based dietary antioxidants are essential for maintaining human health because the human body is unable to produce adequate amounts of antioxidants to protect humans from the persistent threat posed by reactive oxygen species (ROS). Considering the health-protective effect of natural antioxidants and their diverse phytochemical nature, it is expected that plant extracts rich in antioxidant activity and exhibiting anti-infective activity against bacteria will be an advantage to exploit in disease treatment (Muscolo et al., 2024). Therefore, it is expected that systemic screening and evaluation of the rich diversity of Indian medicinal plants using targeted approaches may provide solutions to many health-related issues (Ahmad et al., 2021).

*Murraya koenigii*, commonly known as curry leaf or kari patta in different regions of India, belongs to the Family Rutaceae, which includes about 150 genera and more than 1,300 species, out of which India contributes 71 species. It is commonly used as a flavoring agent in different types of food products and is native to India, Sri Lanka, and other East Asian nations. Many chemical components of various carbazole alkaloids and other significant metabolites, from various parts of the *M. koenigii* plant, have been linked to the medicinal properties of *M. koenigii* (Dahlia et al., 2017). For example, its bark, roots, and leaves can be produced as tonics to induce digestion and have anti-flatulent activity. Following decoction, the leaves acquire a bitter flavor and exhibit antipyretic properties. The leaves and roots have anti-inflammatory and anti-itching properties. They may also be used as an analgesic, a remedy for piles, a heat-reducing agent, and a thirst quencher. They are also helpful in the treatment of blood problems and leukoderma. A paste produced by boiling the green leaves in milk can be used to treat toxic bites and eruptions, while the raw leaves can be used as a treatment for diarrhea (Balakrishnan et al., 2020). Extracts of *M. koenigii* have produced alkaloids, flavonoids, terpenoids, and polyphenols from its leaves, roots, stem bark, fruits, and seeds. It has a substantial amount of important antioxidant phytochemicals, making it suitable for medicinal use, food flavoring, and spicing condiments (Nandy and Das, 2023).

According to our earlier reports, Indian medicinal plants are abundant in new compounds with anti-infective and antioxidant properties (Haris and Ahmad, 2024a). The anti-infective and antioxidant properties of *M. koenigii*, based on its fractions, are discussed in this work and the method of action of phytocompounds using *in silico* research to determine how they might interact with QS and proteins linked to biofilms.

## 2 Materials and methods

### 2.1 Plant material collection

The leaves of *M. koenigii* were gathered locally in Aligarh, Uttar Pradesh, India. The plant biologist at Department of Botany, Aligarh Muslim University, Aligarh recognized the plant material. After being cleaned of dust and debris, the leaves were air-dried in the shade. The departmental repository receives the voucher specimen of the sample (MZK-MK-19/20).

### 2.2 Chemicals

The study used analytical grade organic solvents, chemicals, and reagents. The Azocasein and triphenyl tetrazolium chloride (TTC) were obtained from Sigma Aldrich, USA, and SRL Pvt. Ltd., while microbiological media (LB Agar) and orcinol were obtained from Hi-Media, India. Microbiological media (LB Agar) and orcinol were obtained from Hi-Media, India. All organic solvents, chemicals, and reagents were of analytical grade.

### 2.3 Preparation of methanolic plant extract

The methanolic leaf extract of *M. koenigii* was prepared as mentioned previously (Zahin et al., 2010) with little modification (Haris and Ahmad, 2024a) by soaking 500 grams of dried and powdered plant material in 2.5 L of methanol for 5 days. The extract was filtered, concentrated, and stored at 4 °C for future use.

### 2.4 Bacterial strain and growth requirements

In this study, we used our laboratory isolate *S. marcescens* (Accession no: PP157584), *P. aeruginosa* PAO1 (provided by Prof. R. J. C. McLean, Texas State University, USA), and *C. violaceum* 12472 (ATCC, Manassas, VA, USA). All test pathogens were cultivated in Luria Bertani (LB) broth.

### 2.5 Assays used to determine antioxidant activity

#### 2.5.1 DPPH free radical scavenging assay

The alcoholic DPPH solution is reduced to yellow diphenyl-picrylhydrazine in the presence of an antioxidant sample that donates hydrogen as part of this method for evaluating antioxidant activity (Brand-Williams et al., 1995). A UV–VIS spectrophotometer (UV–VIS-2600, Shimadzu) was used to measure the change in colour from deep violet to yellow at a wavelength of 517 nm.

#### 2.5.2 Ferric-reducing antioxidant power assay (FRAP assay)

The procedure of Oyaizu (1986), as given by Gülçin (2009), was used to ascertain the reducing power of *M. koenigii* leaf extract. The foundation of this technique is the antioxidant samples’ reduction of the ferric/ferricyanide complex to a ferrous state.

### 2.6 Fractionation of the methanolic extract of *Murraya koenigii*

As previously mentioned (Haris and Ahmad, 2024a; Khan et al., 2018), 400 mL of lukewarm distilled water was combined with 20 g of methanolic extract that had been dissolved in 100 mL of methanol. The solution, as mentioned earlier, was mixed with 1 liter of n-hexane and agitated firmly in a separating funnel. The conditions mentioned above were used to collect and evaporatively dry the n-hexane organic phase. Chloroform and ethyl acetate were then used to extract the aqueous phase. After filtering, the remaining aqueous layer was lyophilised using a Scanvac Coolsafe 110–4 Pro. DMSO was used to reconstitute various fractions for later use.

### 2.7 Determination of minimum inhibitory concentration (MIC) of *Murraya koenigii* extract/fractions against bacterial strains

MIC of plant extract/fractions against bacterial strains was determined by the micro broth dilution method, using the specific dye Triphenyl Tetrazolium Chloride (TTC) as an indicator of growth as described by Eloff (1998). Briefly, to make several treatments with different concentrations, 10 μL of the plant extract/fractions was added to 190 μL of nutrient broth in a 96-well microtitre plate and then two-fold diluted to subsequent wells. The leftover 100 microliter (100 μL) of broth from the last well was disposed of, and an inoculum of 100 μL of a diluted culture (1:50) of a distinct log phase bacteria was introduced. The microtitre plate was incubated overnight at the respective optimum growth temperatures of the bacterium. Each well received 20 microlitres (20 μL) of TTC (2 mg/mL), and the plate was incubated for 30 min in the dark at 37 °C. A colour change in the wells was investigated. While no colour change indicated no bacterial growth, the progression of pink to red colour indicated the presence of actively growing cells. Wells showing no change in colour was spotted on Nutrient Agar plates to verify the growth inhibition.

### 2.8 Methods for assessing anti-QS activity of *Murraya koenigii* leaf extract/fractions

#### 2.8.1 Growth curve analysis

The effect of sub-MICs of MKCF on cell growth kinetics was determined. Each bacterial strain was inoculated to 25 mL LB broth with or without different sub-MICs of the fraction, and the OD at 600 nm was monitored at regular intervals of 2 h till 20 h.

#### 2.8.2 Inhibition of violacein in *Chromobacterium violaceum* 12,472

The previously described standard methodology was used to qualitatively evaluate the violacein inhibitory action of the plant extract/ active fraction (McLean et al., 2004; Haris and Ahmad, 2024b). Five milliliters of LB soft agar (0.5% w/v agar) containing *C. violaceum* 12,472 was overlaid on LB agar plates and let the plates kept standing for 20 min. Sterile discs (8 mm) impregnated with varying concentrations of the plant extract/fractions were mounted on the solid media. For 24 h, plates were left incubated at 30 °C and the pigment inhibition in the form of a halo zone around the discs was recorded. Growth inhibition, in the form of clear zones around the impregnated disc, if present was also monitored. The results were expressed in diameter (mm) of pigment inhibition or growth inhibition.

Quantitative evaluation of violacein inhibition in the presence of plant extract/active fraction were also carried out as previously described (Haris and Ahmad, 2024b; Taganna et al., 2011). *C. violaceum* 12,472 with and without different sub-MICs of plant extract was cultured in liquid LB medium at 30 °C for 24 h. Following the incubation, 1 mL of vortexed broth was centrifuged at 12,000 rpm for 10 min. In 1 mL of DMSO, the pellet was reconstituted and vortexed for 5 min to solubilize the cell-bound violacein. The solution was again centrifuge to remove the bacterial cells and the OD_585_ of cell-free DMSO solution was acquired for violacein.

#### 2.8.3 Pyocyanin production

The pyocyanin assay was performed in pseudomonas broth (PB) medium (20 g/L peptone, 1.4 g/L MgCl_2_, and 10 g/L K_2_SO_4_) as this medium enhances the production of pyocyanin by the described procedure (Essar et al., 1990). Briefly, *P. aeruginosa* PAO1 was cultured in PB medium in the absence and presence of varying sub-MICs of MKCF for 18 h. A 5 mL supernatant was extracted with 3 mL of chloroform, and the aqueous phase was discarded. The organic phase was reextracted into 1.2 mL of HCl (0.2 N). The absorbance of the pink or deep red aqueous phase was recorded at 520 nm. The concentration of pyocyanin is expressed in μg/ml, which was obtained by multiplying the OD_520_ by 17.072.

#### 2.8.4 Pyoverdin production

The previous standard procedure was used to measure the pyoverdin levels spectrophotometrically (Ankenbauer et al., 1985). Briefly, to produce a cell-free supernatant, overnight developed cultures of *P. aeruginosa* PAO1 in the presence and absence of sub-MICs of MKCF were centrifuged. 900 μL of 50 mM Tris–HCl (pH 7.4) were combined with 100 μL of supernatant. Using an RF-5301PC spectrofluorometer (Shimadzu, Japan), the sample’s fluorescence emission signal was excited at 400 nm and measured at 460 nm.

#### 2.8.5 Proteolytic activity

The proteolytic activity of the bacterial strains under the effect of different sub-MICs of the MKCF was determined by azocasein degradation assay as previously described (Husain et al., 2017). Briefly, 100 μL cell-free supernatant of untreated and treated cultures were mixed with 1 mL of 0.3% (w/v) azocasein (containing 0.5 mM CaCl_2_ in 0.05 M Tris- HCl, pH 7.5), and the reaction mixture was incubated for 15 min at 37 °C. Five hundred microlitres of trichloroacetic acid (10% w/v) was added to terminate the reaction and then centrifuged at 12000 rpm for 10 min. The absorbance of the supernatant was recorded at 400 nm.

#### 2.8.6 Rhamnolipid production

The previously stated orcinol procedure was used to execute the rhamnolipid test (Haris and Ahmad, 2024a; Husain et al., 2017). In brief, *P. aeruginosa* was cultivated for 18 h at 37 degrees Celsius both in the absence and presence of sub-MICs of MKCF, and the cell supernatant was collected by centrifugation. Six hundred microliter diethyl ether was combined with 300 microlitres of cell-free supernatant from cultures. The mixture was vortexed for 1 min. A 100 microlitre solution of deionized water was used to reconstitute the organic phase after it had been separated and evaporated to dryness at 37 degrees Celsius. A hundred microliter of each sample was added with 900 μL of an orcinol solution. For 30 min, the mixture was heated to 80 °C. After cooling the sample for 15 min at room temperature, absorbance at 421 nm was measured.

#### 2.8.7 Motility assay

In order to assess the swimming motility, LB plates (0.3% agar) were spotted with 5 microlitres of the bacterial culture that had been cultivated overnight, and the plates were allowed to dry at room temperature (Haris and Ahmad, 2024a). The control group consisted of plates devoid of MKCF. After 18 h of incubation, the plates were examined, and the swimming zone was measured by the transparent ruler in millimeters (mm).

#### 2.8.8 Prodigiosin production

In Luria-Bertani media, prodigiosin pigment was evaluated using the established procedure as described earlier (Haris and Ahmad, 2024b; Slater et al., 2003). In brief, for 18 h at 30 °C, *S. marcescens* was cultivated both in the absence and presence of sub-MICs of active plant extracts/fractions. The bacterial cells were pelleted by centrifuging two milliliters of the growing culture at 10,000 rpm for 5 min. A rigorous vortexing process lasting 5 min was used to dissolve the pellet in 1 mL of an acidified ethanol solution. In order to eliminate debris, the sample was centrifuged once more for 5 min at a speed of 13,000 rpm. Using a UV-2600 spectrophotometer, the absorbance was measured at 534 nm.

#### 2.8.9 Exoprotease activity

As previously mentioned, the azocasein degradation technique was employed to evaluate *S. marcescens’s* exoprotease activity (Salini and Pandian, 2015). Briefly, *S. marcescens* was grown for 18 h at 30 degrees Celsius with and without sub-MIC levels of MKCF. 100 microliters of the supernatant that was left over after centrifuging the culture was mixed with one milliliter of 0.3% (w/v) azocasein. The reaction mixture was shaken and then incubated for 15 min at 37 °C. After adding 0.5 mL of ice-cold TCA to terminate the reaction, the insoluble azocasein was removed by centrifugation. At 400 nm, the absorbance was taken with a UV-2600 spectrophotometer.

### 2.9 Inhibition of biofilm formation

#### 2.9.1 Crystal violet method

MKCF’s quantitative assessment of biofilm inhibition was evaluated on a 96-well microtitre plate using the previously described crystal violet technique (O’Toole and Kolter, 1998). Following an overnight incubation period, bacterial cultures with various sub-MICs of MKCF were introduced to the wells containing LB media. After three rounds of cleaning with sterile phosphate buffer to eliminate extra broth and planktonic cells, the wells were left to air dry for 20 min. The biofilms were gently rinsed three times to eliminate the stain after being stained for 15 s with 200 μL of crystal violet. The crystal violet affixed to the biofilm was extracted using 200 microliters of 90% ethanol, and the absorbance at 620 nm was measured using a microplate reader (Thermo Scientific Multiskan EX, UK).

#### 2.9.2 Biofilm light microscopy

Biofilms on glass coverslips were inhibited using the previously described method (Sybiya Vasantha Packiavathy et al., 2012). Briefly, 60 μL overnight-grown cultures of the bacterial pathogens were seeded into a 24-well culture plate containing 3 mL of culture media. Furthermore, sterile glass coverslips with the highest sub-MICs of MKCF were placed in the wells. After a 24-h incubation period, the loosely attached cells were rinsed three times with sterile phosphate buffer solution and allowed to air dry for 20 min. Slides were left to air dry for half an hour after being stained with crystal violet solution. A light microscope (Olympus BX60, Model BX60F5, Olympus Optical Co., Ltd., Japan) equipped with a colour VGA camera (Sony, Model no. SSC-DC-58AP, Japan) was used to view the biofilms.

#### 2.9.3 Biofilm microscopic analysis using scanning Electron microscope and confocal laser scanning microscope

Biofilms formed on coverslips, as mentioned before. Unbound bacterial cells were removed after being cleaned with sterile phosphate buffer and fixed with 2.5% glutaraldehyde. The adherent cells and biofilms were then dried for 10 min using an ethanol gradient. The slides were air-dried and gold-coated before visualization. A JEOL-JSM 6510 LV was utilized by the University Sophisticated Instrumentation Facility (USIF), AMU, Aligarh, to take the SEM micrographs.

For CLSM, biofilms were grown on glass surfaces using the same procedure as previously mentioned. The biofilms were then stained for 20 min with acridine orange (0.1%). The images were taken at USIF, AMU, Aligarh, with a Zeiss LSM780.

### 2.10 Phytochemical examination of MKCF leaf extract

#### 2.10.1 TLC-based detection of major phytochemical groups

Different fractions of active plant extracts were preliminarily tested for the presence of a common class of plant compounds, such as terpenoids, flavonoids, and alkaloids, using thin-layer chromatography (TLC) according to the previously reported standard procedure (Wagner and Bladt, 1996; Harborne, 1998). All extracts were dried and redissolved in methanol. The samples were then spotted on TLC plates using the Pasteur pipette. The chromatograms of plant extract were developed using ethyl acetate, toluene, and formic acid (5:4:1) on TLC Silica 60 F254 plates (Merck, Germany). The details of the phytochemical class, reagents used, band colors observed, and their inference is presented in Supplementary material SI 10.

#### 2.10.2 Gas chromatography- mass spectrometry (GC/MS)

MKCF was subjected to GC/MS analysis for the identification and relative quantification of its phytocompounds. GC 7890A (Agilent Technologies, Santa Clara, CA, USA) systems, along with the Accutof GCv JMST100 mass spectrometer (JEOL India Pvt. Ltd.) were used for the GC–MS analysis. To identify compounds, the observed peak mass spectra were compared to a standard database (the NIST library).

#### 2.10.3 LC-qTOF/MS analysis

To further identify compounds using the previously established approach, MKCF was employed in LC-qTOF/MS analysis (Khan et al., 2018). The Agilent 1,290 infinite UPLC machine (Agilent Technologies, USA) was used to perform chromatographic separation on a C18 column. The spectra were recorded using the quadrupole time-of-flight unit. The Sophisticated Analytical Instrument Facility (SAIF) at the Indian Institute of Technology Bombay (IIT-B), Maharashtra, India, is where the LC-qTOF/MS analysis was conducted. The identification of phytocompounds was done using Agilent Mass Profiler Professional (MPP) software.

### 2.11 *In silico* molecular docking studies

To predict the binding affinity and optimal binding location of phytocompounds with proteins or enzymes involved in QS-mediated virulence factors and biofilms, docking experiments were conducted using AutoDock 4.2.6 and AutoDock Tools (ADT) with Lamarckian genetic algorithm (LGA) (Morris et al., 2009). The 3D structure of phytocompounds, i.e., Ismine (CID: 188957), Murrayazolinol (CID: 180314), Morellin (CID: 71306322), and the 2D structures of 4-O-Methylphorbol 12,13-didecanoate (CID: 119493), Gibberellic acid, methyl ester (CID: 539615), and 7,8-Epoxylanostan-11-ol,3-acetoxy- (CID: 541562), were downloaded from PubChem^1^. All the 2D structures were converted into 3D structures with ACD/Chemsketch software. All the 3D structures in sdf format were converted into pdb files through Open Babel GUI. The crystal structures of receptor proteins (PilY1, LasA, CviR, and EsaI) were obtained in pdb format (PDBID: 3HX6, 3IT7, 3QP5, and 1KZF, respectively) from the RCSB Protein Data Bank^2^. Kollman charges (−3.496 to 3HX6, 10.0 to 3IT7, 13.0 to 3QP5, and −2.248 to 1KZF) were added to receptors using AutoDock tools. The SPDBV program was used to apply a steepest descent energy reduction process that involved 20 steps. The center points for 3HX6 were 8.037, 8.651, and 6.996; for 3IT7, they were 19.199, −3.455, and −5.562; for 3QP5, they were 30.652, 12.059, and −4.797; whereas for 1KZF, these were 29.8340, −0.63076, and 2.6290. The CASTp 3.0 internet server^3^ was used to determine the receptors’ active sites (Tian et al., 2018), and co-crystallized ligands in the receptors’ active site cavity verified these findings. The grid box, which had dimensions of 80 Å, 80 Å, and 80 Å along the x, y, and z axes with a grid spacing of 0.503 Å, was created around the receptors’ active site residues. Docking parameters included a population size of 150, several energy assessments of 25,00,000, and 10 LGA runs. All other parameters were left at their default settings. The conformer with the lowest binding energy was chosen for additional research. Discovery Studio 2021 was used to develop post-docking pictures that showed the suggested binding modes.

### 2.12 Statistical analysis

SigmaPlot software, version 12.3 (Systat Software, Inc., San Jose, CA, USA), was used to perform statistical computations. Every experiment was run in three duplicates. The average values with plus minus standard deviation represent the study’s findings. The t-test was used for comparison between the treatment and control groups. *p* values≤ 0.05 were deemed significant.

## 3 Results

### 3.1 Antioxidant activity of *Murraya koenigii* (leaves)

**Figure 1:**
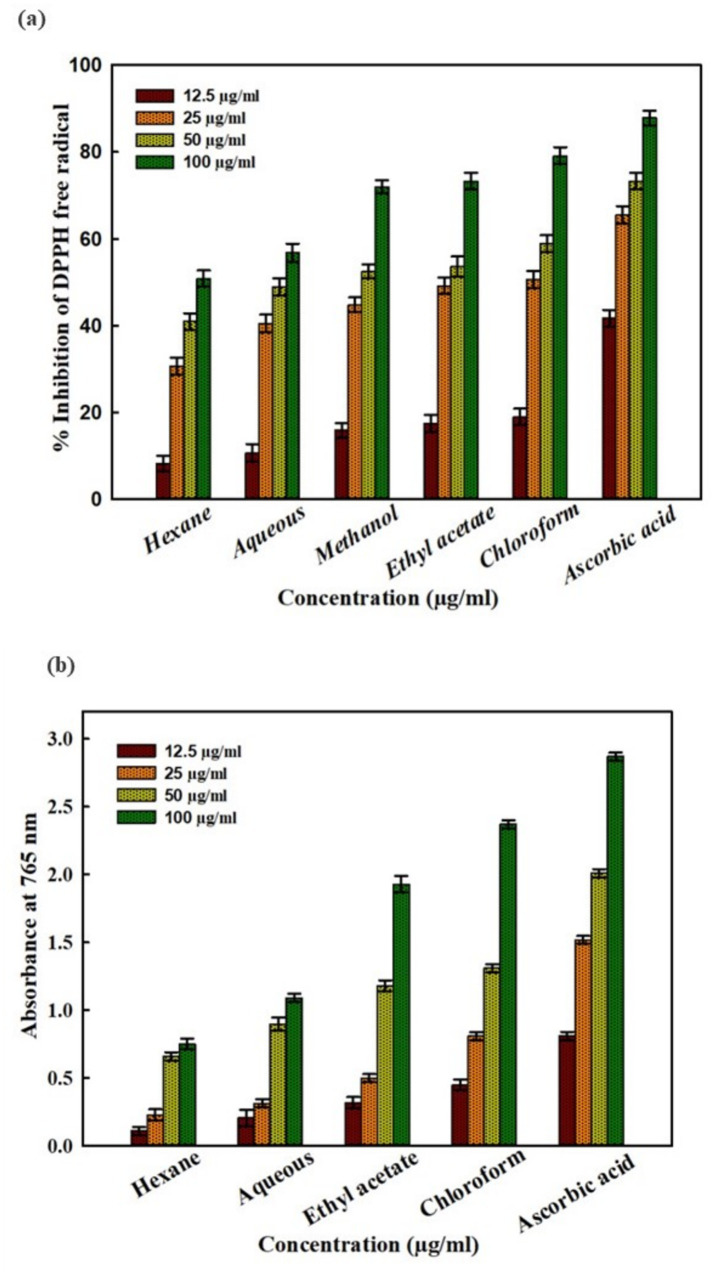
Antioxidant activity of different fractions of *M. koenigii* (leaves). **(a)** Free radical scavenging activity (DPPH assay) of fractions and **(b)** ferric-reducing ability of fractions.

DPPH and FRAP assays were used to further investigate the antioxidant activity of the liquid–liquid extracted fractions of *M. koenigii* (leaves). Different degrees of free radical scavenging activity were demonstrated by fractions of *M. koenigii* (leaves) extract. The hexane fraction exhibited the lowest activity, as seen in Figure 1a, whereas the most active fraction, chloroform, inhibited 79.11% of total DPPH radicals at a concentration of 100 μg/mL. The IC_50_ values for hexane, chloroform, ethyl acetate, and aqueous extracts were determined to be 98.43, 28.59, 30.93, and 45.93 μg/mL, respectively. Similarly, while assessing reducing power, these fractions showed concentration-dependent responses. The ferric-reducing activity was recorded highest for chloroform, followed by the ethyl acetate fraction. The hexane fraction showed the least reducing power, as shown in Figure 1b.

### 3.2 Fractional screening of *Murraya koenigii* (leaves) extracts for anti-QS activity

**Figure 2:**
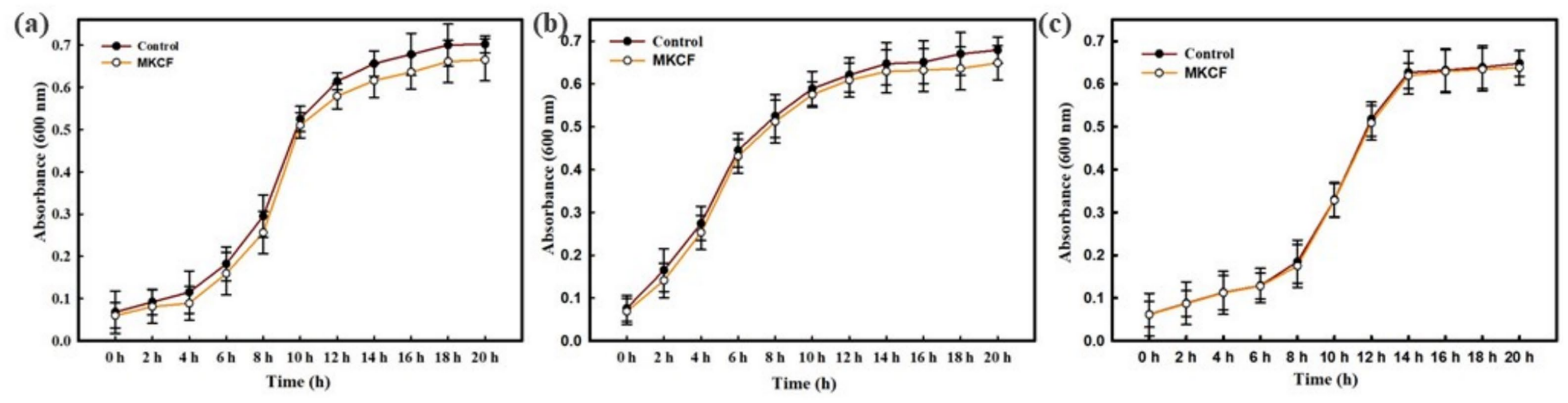
Growth curves of **(a)** *P. aeruginosa* PAO1 **(b)** *C. violaceum* 12,472, and **(c)** *S. marcescens* at the highest tested respective sub-MIC (MIC/2) of MKCF.

The MKCF demonstrated varying levels of MIC values against bacterial pathogens, as depicted in Table 1. At sub-inhibitory concentrations, plant extract/fractions did not show any significant growth inhibitory effect against bacterial pathogens, as shown in Figure 2. All experiments were carried out at their respective sub-MICs.

**Table 1:** Minimum inhibitory concentration (MIC) of MKCF against bacterial pathogens.

Plant / Part used	Fraction	Bacteria		
		*P. aeruginosa* PAO1	*C. violaceum* 12,472	*S. marcescens*
*Murraya koenigii* (Leaves)	Methanol	4	2	2
	Hexane	8	4	8
	Chloroform	2	2	2
	Ethyl acetate	2	2	2
	Aqueous	4	2	4

The MICs presented are in mg/ml. The MIC was determined by micro broth dilution assay using TTC as an indicator dye.

The anti-QS activity of each fraction was further examined against the production of pyocyanin in *P. aeruginosa* PAO1 and violacein in *C. violaceum*. Every experiment was conducted at its corresponding sub-MIC.

#### 3.2.1 Inhibition of violacein in *Chromobacterium violaceum* 12,472

The methanolic extract of *M. koenigii* leaves was fractioned using liquid–liquid partitioning in different solvents, with the chloroform fraction (MKCF) showing the highest violacein inhibition (75.29%), as shown in Table 2, prompting further investigation into its bioactive anti-QS fraction.

**Table 2:** Anti-QS screening of *M. koenigii* leaves extract against *C. violaceum* 12,472.

Plant	Fraction	OD_585nm_	Percent inhibition of violacein production
Untreated control		0.680 ± 0.1	-
*M. koenigii*	Methanol	0.248 ± 0.02^**^	63.52
	Hexane	0.497 ± 0.01^*^	26.91
	Chloroform	0.168 ± 0.02^**^	75.29
	Ethyl acetate	0.232 ± 0.02^**^	65.88
	Aqueous	0.413 ± 0.03^*^	39.26

The data is presented as the average ± SD of three replicates. Percent inhibition was calculated with respect to the control. The violacein inhibition in broth assay was done at the respective ½ × MICs of fractions. ^*^ indicates significance at *p* ≤ 0.05, ^**^ significance at *p* ≤ 0.01 compared to untreated control.

#### 3.2.2 Inhibition of pyocyanin in *Pseudomonas aeruginosa* PAO1

The fractions of *M. koenigii* were also tested for their ability to prevent *P. aeruginosa* PAO1 from producing pyocyanin through QS (Table 3). The chloroform fraction of *M. koenigii* (MKCF) was more promising than other fractions, demonstrating 75.35% pyocyanin inhibition. Other fractions of *M. koenigii* following the chloroform fraction were ethyl acetate (70.05%) > aqueous (49.14%) > hexane (24.49%).

**Table 3:** Anti-QS screening of *M. koenigii* leaves extract against *P. aeruginosa* PAO1.

Plant	Fraction	Pyocyanin (μg/ml)	Percent inhibition of pyocyanin production
Untreated control		6.98 ± 0.36	-
*M. koenigii*	Methanol	2.18 ± 0.31^***^	68.76
	Hexane	5.27 ± 0.17^*^	24.49
	Chloroform	1.72 ± 0.18^***^	75.35
	Ethyl acetate	2.09 ± 0.24^***^	70.05
	Aqueous	3.55 ± 0.45^***^	49.14

The data is presented as the average ± SD of three replicates. Percent inhibition was calculated with respect to the control. Quantitative assessment of pyocyanin production was done at the respective ½ × MICs of fractions. ^*^ indicates significance at *p* ≤ 0.05, ^***^ significance at *p* ≤ 0.005 compared to untreated control.

### 3.3 Effect of MKCF on virulence factors controlled by QS

#### 3.3.1 Inhibition of violacein in *Chromobacterium violaceum*

**Figure 3:**
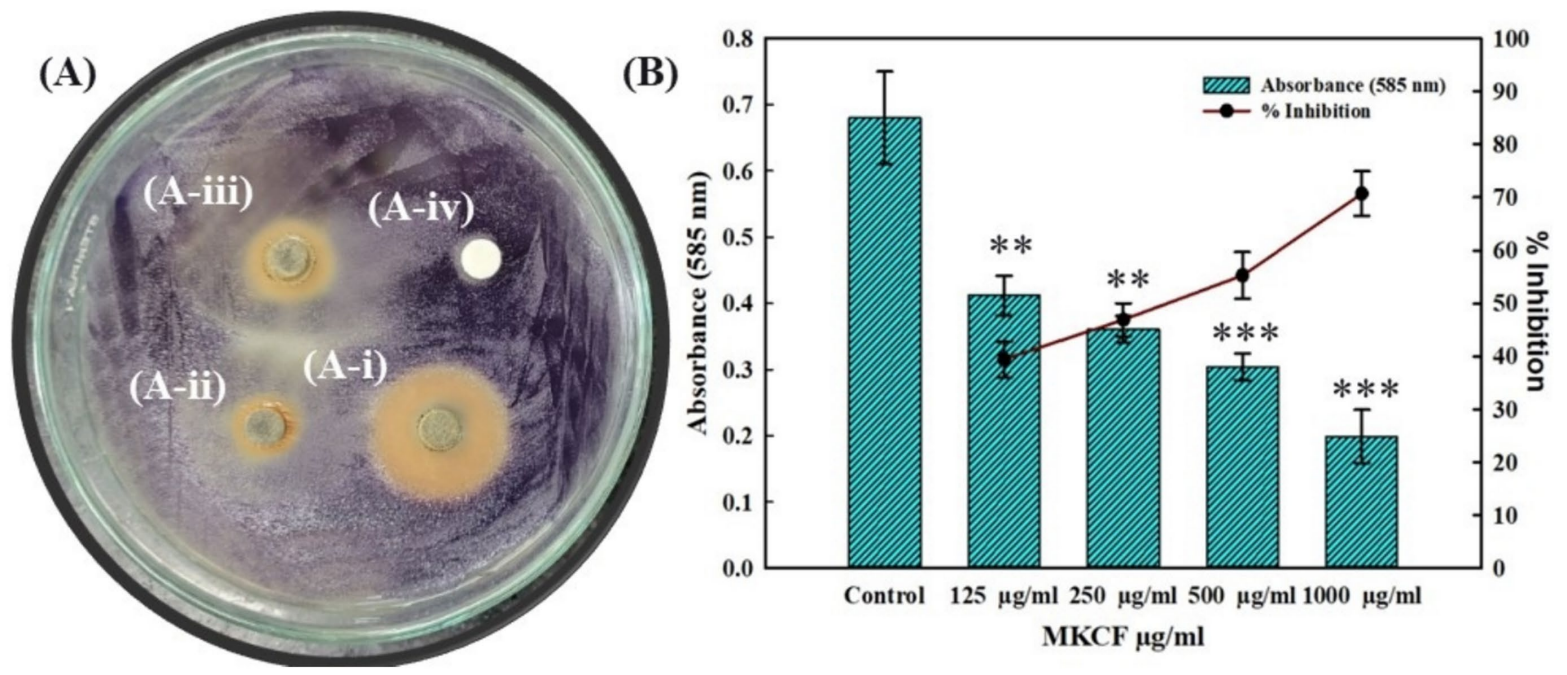
**(A)** Effect of MKCF on the violacein pigment production by *C. violaceum* 12,472 by disc diffusion. A-i: *M. koenigii* (chloroform: 1 mg/mL); A-ii: *M. koenigii* (chloroform: 0.5 mg/mL); A-iii: *M. koenigii* (methanolic: 1 mg/mL); A-iv: DMSO (control). **(B)** Quantitative analysis of violacein inhibition in *C. violaceum* in the absence and presence of MKCF. Data are represented as mean values of triplicate readings, and the bar is the SD. The percent inhibition is shown on the secondary y-axis. ** indicates *p* ≤ 0.01 with respect to control; *** indicates *p* ≤ 0.005 with respect to control.

MKCF was primarily evaluated for its effect on violacein pigment production by *C. violaceum* 12,472 using the disc diffusion method at concentrations of 500 and 1,000 μg/mL per disc. Methanolic crude extract and most antioxidant-active chloroform fraction exhibited varying levels of pigment inhibition at the tested concentrations (Figure 3A). At the highest tested concentration (1,000 μg/mL) impregnated disc, MKCF inhibited violacein production more effectively than the methanolic extract. Violacein inhibition was also quantitatively estimated when sub-MICs (125–1,000 μg/mL) of MKCF were present. Figure 3B illustrates how the most active chloroform fraction inhibits the formation of violacein. The findings showed that the violacein pigment was reduced by 39.41, 46.91, 55.29, and 70.73% in the presence of 125, 250, 500, and 1,000 μg/mL MKCF, respectively. Azithromycin (2 μg/mL) was used as positive control (data not shown).

#### 3.3.2 Effect of MKCF on the virulence factors of *Serratia marcescens* controlled by QS

MKCF was also tested for anti-QS property against *S. marcescens,* and the results are shown in Table 4. Comparing the pigment levels to the untreated control, the addition of 125, 250, 500, and 1,000 μg/mL MKCF decreased them by 13.20, 30.18, 49.05, and 62.26%, respectively. Protease activity was shown to be affected by MKCF in a dose-dependent manner and inhibited by 68.75% when 1,000 μg/mL MKCF was present. *S. marcescens* was examined for its ability to swarm over agar plates, supplemented with MKCF. When 1,000 μg/mL MKCF was present, the swarming motility was 76.03% lower than the control.

**Table 4:** Effect of sub-MICs of *M. koenigii chloroform* fraction (MKCF) on inhibition of virulence factors in *S. marcescens*.

Fraction concentration (μg/ml)	Virulence factor production		
	Prodigiosin^a^	Protease activity^b^	Swarming motility^c^
Untreated control	0.53 ± 0.02	0.64 ± 0.02	89.00 ± 1.00
125	0.46 ± 0.02* (13.20)	0.54 ± 0.03* (15.62)	72.00 ± 3.00*** (19.10)
250	0.37 ± 0.03** (30.18)	0.42 ± 0.03*** (34.37)	60.00 ± 2.00*** (32.58)
500	0.27 ± 0.03*** (49.05)	0.32 ± 0.05*** (50.00)	38.66 ± 2.08*** (56.56)
1,000	0.2 ± 0.04** (62.26)	0.2 ± 0.03*** (68.75)	21.33 ± 2.51*** (76.03)

a Prodigiosin were expressed as the absorbance at 534 nm.

b Total protease activity is expressed as the absorbance at 400 nm.

c Swarming motility is expressed as swarm diameter in mm.

The data represent the mean value of three independent experiments. *significance at *p* ≤ 0.05, ** significance at *p* ≤ 0.01, *** significance at *p* ≤ 0.005. The value in parentheses represents the percent reduction over the untreated control.

#### 3.3.3 Effect of MKCF on the virulence factors of *P. aeruginosa* controlled by QS

The findings in Table 5 demonstrate MKCF’s *in vitro* anti-QS activity against *P. aeruginosa* PAO1’s virulence factors, which showed significant inhibition in a concentration-dependent manner. The pyocyanin level in untreated *P. aeruginosa* was 6.98 ± 0.22 μg/mL, and it dropped in a concentration-dependent way following MKCF treatment. Pyocyanin production was inhibited by 9.45, 19.19, 45.98, and 64.75% after treatment with 125, 250, 500, and 1,000 μg/mL MKCF, respectively. Likewise, in the presence of 1,000 μg/mL MKCF, the pyoverdin fluorescence was maximally decreased by 54.39% in a concentration-dependent manner. Protease activity showed a similar downward trend, decreasing by 56.66% at the highest measured sub-MIC (1,000 μg/mL) in comparison to the untreated control. In comparison to the untreated control, the rhamnolipid content also decreased dose-dependently, with the concentration lowered by 55.88% at the highest measured sub-MIC (1,000 μg/mL). There was a 56.28% reduction in swarming motility in the presence of 1,000 μg/mL MKCF.

**Table 5:** Effect of sub-MICs of *M. koenigii* chloroform fraction (MKCF) on inhibition of virulence factors in *P. aeruginosa* PAO1.

Fraction concentration (μg/ml)	QS-regulated Virulence factors production				
	Pyocyanin^a^	Pyoverdin^b^	Exoprotease^c^	Rhamnolipid^d^	Swimming motility^e^
Untreated control	6.98 ± 0.22	750.3 ± 30.3	0.90 ± 0.02	1.02 ± 0.04	66.33 ± 3.51
125	6.32 ± 0.21* (9.45)	657.3 ± 21.4* (12.39)	0.75 ± 0.03* (16.66)	0.82 ± 0.03* (19.60)	61.00 ± 1.00* (8.03)
250	5.64 ± 0.45** (19.19)	560.5 ± 34.2* (25.29)	0.61 ± 0.05** (32.22)	0.76 ± 0.04* (25.49)	53.66 ± 0.57* (19.10)
500	3.77 ± 0.30*** (45.98)	472.1 ± 43.2** (37.07)	0.46 ± 0.04*** (48.88)	0.60 ± 0.02*** (41.17)	43.33 ± 3.05*** (34.67)
1,000	2.46 ± 0.34*** (64.75)	342.2 ± 18.4*** (54.39)	0.39 ± 0.06*** (56.66)	0.45 ± 0.06** (55.88)	29.00 ± 1.00*** (56.28)

a Pyocyanin concentrations were expressed as μg/ml of culture supernatant.

b Pyoverdin production is expressed as relative fluorescence.

c Exoprotease activity is expressed as the absorbance at 400 nm.

d Rhamnolipid is expressed as the absorbance at 421 nm.

e Swimming motility is expressed as diameter of swarm in mm.

The data represent the mean value of three independent experiments. *significance at *p* ≤ 0.05, ** significance at *p* ≤ 0.01, *** significance at *p* ≤ 0.005.

The value in parentheses represents the percent reduction over untreated control.

### 3.4 Effect of MKCF on biofilm formation

Glass coverslips and a 96-well microplate experiment were used to examine the impact of MKCF on the biofilm formation of bacterial pathogens.

#### 3.4.1 Effect of MKCF on biofilm formation of bacterial pathogens

Table 6 shows that the tested fraction inhibited the production of biofilms in a dose-dependent manner. A 1000 μg/mL concentration of MKCF decreased *P. aeruginosa* biofilms by 66.07%. Comparing the biofilms of *C. violaceum* and *S. marcescens* to the untreated control, 1,000 μg/mL MKCF decreased them by 76.62 and 64.83%, respectively.

**Table 6:** Effect of MKCF on biofilm formation against test bacterial pathogens.

Fraction concentration (μg/ml)	OD_620nm_		
	*C. violaceum* 12,472	*P. aeruginosa* PAO1	*S. marcescens*
Control	0.77 ± 0.1	1.12 ± 0.07	0.91 ± 0.09
125	0.56 ± 0.11*** (27.27)	0.92 ± 0.10*** (17.85)	0.79 ± 0.10*** (13.18)
250	0.49 ± 0.11*** (36.36)	0.81 ± 0.09* (27.67)	0.62 ± 0.07* (31.86)
500	0.33 ± 0.06*** (57.14)	0.60 ± 0.11*** (46.42)	0.46 ± 0.09* (49.45)
1,000	0.18 ± 0.08*** (76.62)	0.38 ± 0.12*** (66.07)	0.32 ± 0.08* (64.83)

Biofilm formation is expressed as OD _620_ after incubation with crystal violet.

The data represent the mean value of three independent experiments. *significance at *p* ≤ 0.05, ** significance at *p* ≤ 0.01, *** significance at *p* ≤ 0.005.

The value in parentheses represents the percent reduction over control.

#### 3.4.2 Microscopic studies on the antibiofilm effect of MKCF

**Figure 4:**
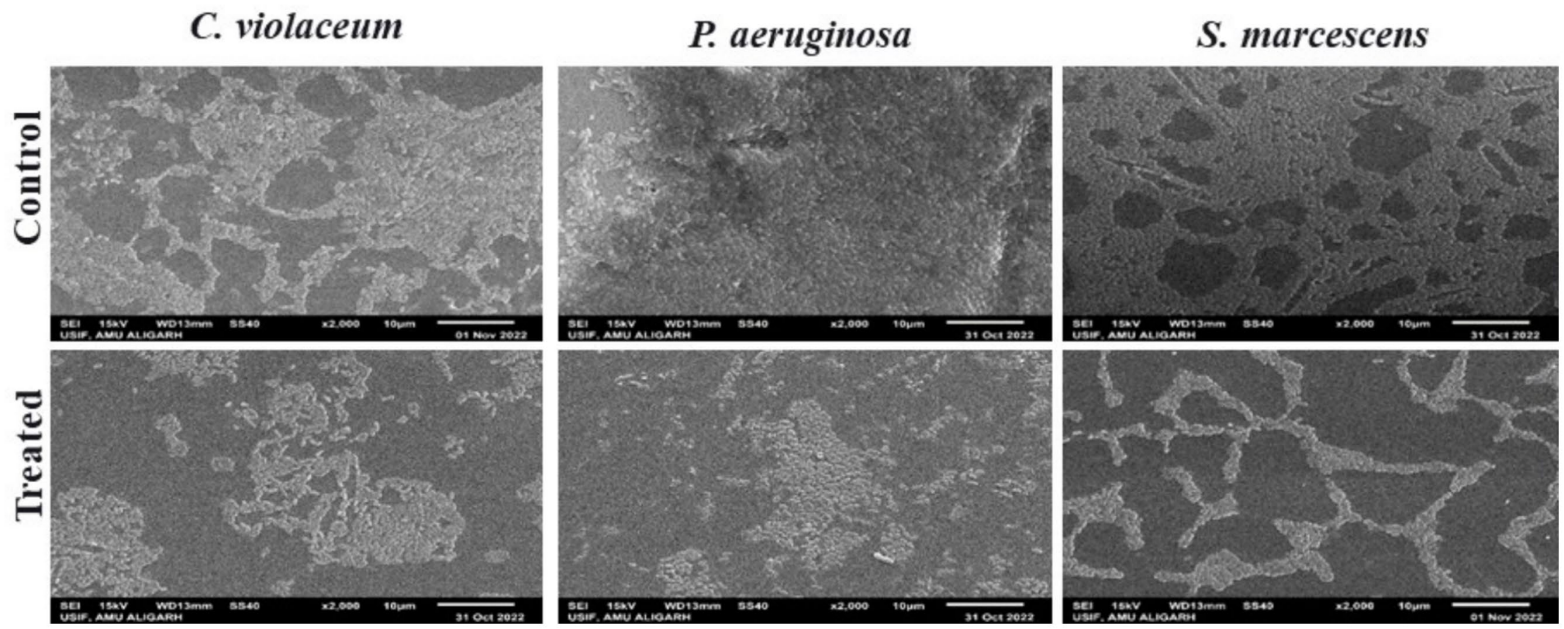
Scanning electron micrographs of *C. violaceum*, *P. aeruginosa,* and *S. marcescens* biofilm in the absence and presence of sub-MIC (1,000 μg/mL) of MKCF.

**Figure 5:**
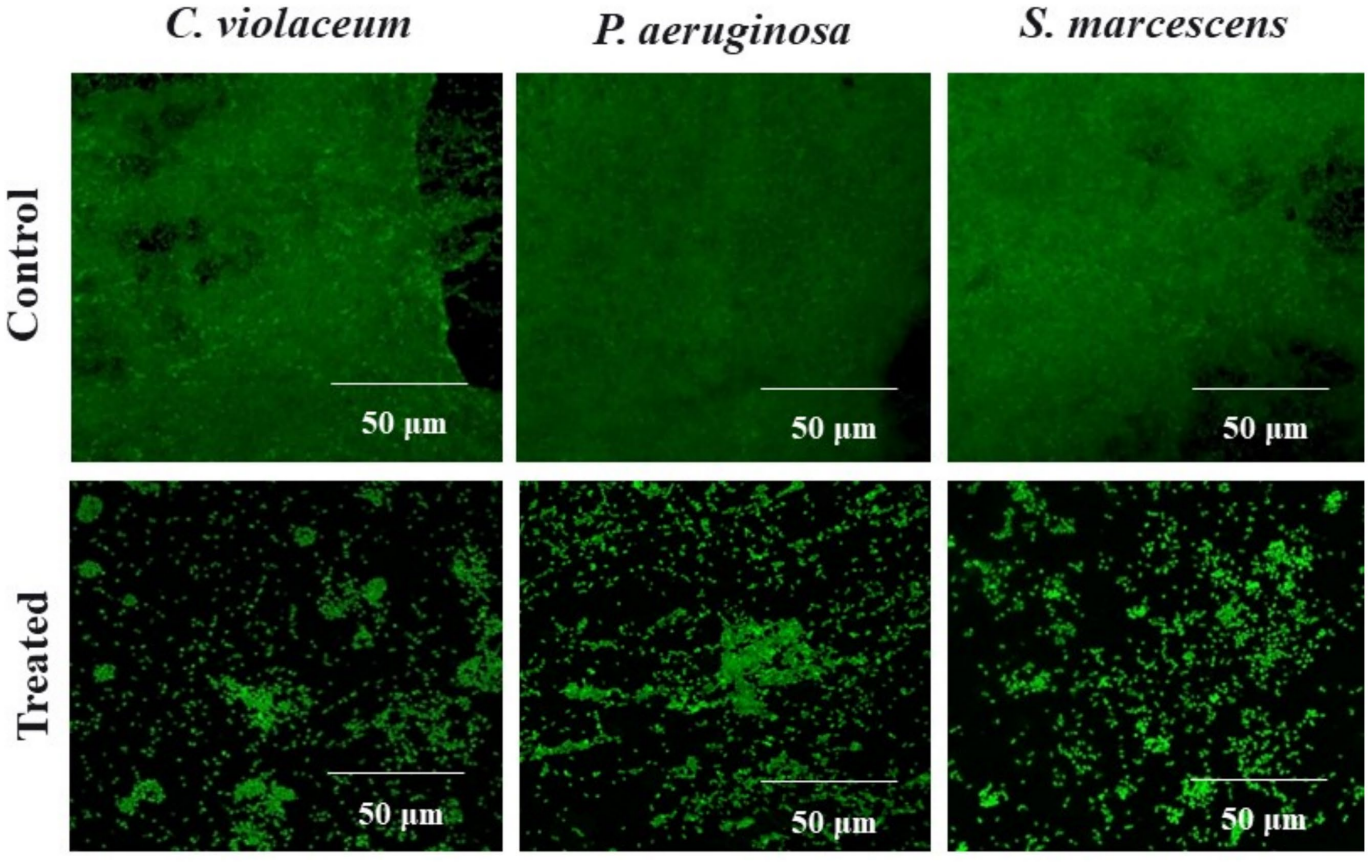
Confocal laser scanning microscopic images of *C. violaceum*, *P. aeruginosa* and *S. marcescens* biofilm in the absence and presence of sub-MIC (1,000 μg/mL) of MKCF.

Following a quantitative evaluation of plant extracts’ ability to suppress biofilm development using the microbroth dilution experiment, the inhibition of biofilm formation on a glass coverslip was examined. In the untreated control, bacterial cells formed a thick and dense biofilm on the glass surface, as shown in the light microscopic pictures of bacterial biofilm (Supplementary material SI 1). As indicated by the scattered cells, treatment with 1,000 μg/mL MKCF decreased the bacterial cells’ tendency to aggregate on the glass surface. Subsequent SEM examination revealed that the untreated control showed thick and compact biofilm growth on the glass surface that resembled exopolysaccharides (EPS) (Figure 4). In comparison to the untreated control, treatment with the highest sub-MIC of MKCF produced dispersed bacterial cells and fewer microcolony clumps. A thick carpet-like structure generated by bacterial pathogens on the surface of the glass control (untreated) was visible in the CLSM images. Nevertheless, the presence of the highest sub-MIC, i.e., 1,000 μg/mL MKCF, decreased the production of biofilms (Figure 5).

### 3.5 Phytochemical analysis of the chloroform fraction of *Murraya koenigii*

**Figure 6:**
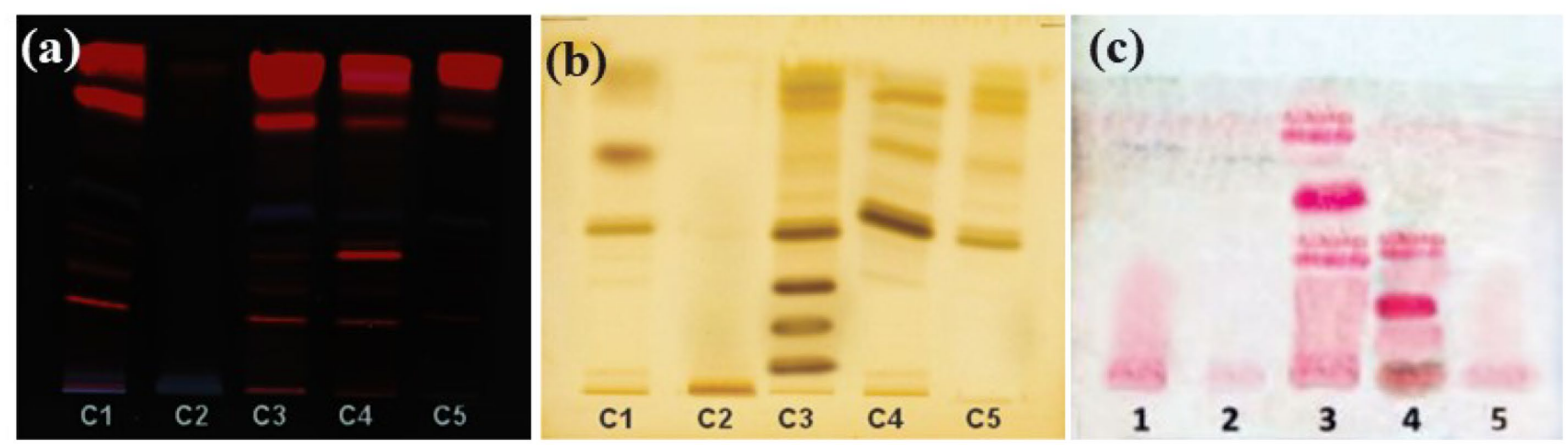
TLC chromatograms of different fractions of *M. koenigii* extract. Band: (1) Methanol, (2) Hexane, (3) Chloroform, (4) Ethyl acetate, and (5) Aqueous fractions. **(a)** Natural product reagent plus UV-365 nm (flavonoids detection). **(b)** Drangendorff’s reagent plus visible light (alkaloid detection). **(c)** Vanillin-sulfuric acid reagent plus visible light (terpenoids/phenyl propenoid detection).

TLC-based detection of the phytocompounds class revealed the presence of alkaloids, terpenoids, and flavonoids in the most bioactive chloroform fraction, as well as the ethyl acetate fraction, as shown in Figure 6. Only alkaloids and flavonoids were found in the aqueous fraction. Terpenoids were absent in the aqueous fraction. However, none of the tested phytochemical class of compounds could be detected by TLC in the hexane fraction of *M. koenigii* (Table 7).

**Table 7:** Detection of major phytochemical classes in different fractions of *M. koenigii* extract by TLC.

Name of the plant	Fractions	Major phytocompounds group detected		
		Alkaloids	Flavonoids	Terpenoids
*M. koenigii*	Hexane	−	−	−
	Chloroform	+	+	+
	Ethyl acetate	+	+	+
	Aqueous	+	+	−

Table 8 lists the phytocompounds identified in MKCF by GC/MS analysis, and Supplementary material SI 2 displays the chromatogram. Some major compounds found with high percent peak area are Gibberellic acid, methyl ester (7.16%), 7,8-Epoxylanostan-11-ol, 3-acetoxy- (5.74%), 1′,1’-Dicarboethoxy-1β,2β-dihydro-3’H-cycloprop(1,2)cholesta-1,4,6-trien-3-one (3.73%), 3,9- Epoxypregn-16-ene-14,20-diol,7,11,18-triacetoxy-3-methoxy- (2.32%), respectively.

**Table 8:** Major phytocompounds of MKCF identified by GC/MS analysis.

Peak	Area %	Compound name	Compound class
2	1.74	8,9-Seco-3,19-epoxyandrostane-8,9-dione, 17-acetoxy-3-methoxy-4,4-dimethyl-	Ketals
3	7.16	Gibberellic acid, methyl ester	Diterpenoids
4	2.32	3,9- Epoxypregn-16-ene-14,20-diol,7,11,18-triacetoxy-3-methoxy-	Steroids
5	10.16	4-O-Methylphorbol 12,13-didecanoate	Diterpenoids
7	5.74	7,8-Epoxylanostan-11-ol, 3-acetoxy-	Triterpenoids
10	7.83	2- Oleo-palmitostearin	Fatty acid conjugates
12	3.73	1′,1’-Dicarboethoxy-1β,2β-dihydro-3’H-cycloprop(1,2)cholesta-1,4,6-trien-3-one	Fatty acid conjugates

Table 9 lists the main phytocompounds found by LC-qTOF/MS analysis, and Supplementary material SI 3 displays the chromatogram. The most prevalent compounds were murrayazolinol, ismine, morellin, bismahanine, phytosphingosine, etc.

**Table 9:** Major phytocompounds of MKCF identified by LC-qTOF/MS analysis.

Compounds	Molecular formula	Molecular mass	Chemical class
Retrofractamide D	C21 H27 N O3	341.1994	Benzodioxoles
Piperolein B	C21 H29 N O3	343.2138	Benzodioxoles
C16 Sphinganine	C16 H35 N O2	273.2659	Sphingolipid
Phytosphingosine	C18 H39 N O3	317.2915	Sphingolipid
Cinncassiol C1	C20 H28 O7	380.1849	Terpene glycosides
17-Hydroxylinolenic acid	C18 H30 O3	294.2183	Fatty acids
Ismine	C15 H15 N O3	257.1041	Alkaloids
Ganolucidic acid A	C30 H44 O6	500.3139	Triterpenoids
Murrayazolinol	C23 H25 N O2	347.1891	Phenanthridines and derivatives
Goshonoside F5	C32 H54 O13	646.3611	Diterpene glycosides
Corchorifatty acid F	C18 H32 O5	328.2248	Lineolic acids and derivatives
Prodelphinidin A2 3′-gallate	C37 H28 O18	760.1349	Biflavonoids
Gravelliferone	C19 H22 O3	298.1604	Hydroxycoumarin
Capsoside A	C33 H58 O15	694.3789	Glycosyl diacylglycerols
Bismahanine	C46 H48 N2 O4	692.3656	Alkaloid
Vignatic acid B	C27 H41 N3 O7	519.2907	Cyclic peptides
Lyciumoside III	C32 H56 O13	648.375	Diterpene glycosides
Morellin	C33 H36 O7	544.2456	Organic disulfides

### 3.6 *In silico* molecular docking studies

**Figure 7:**
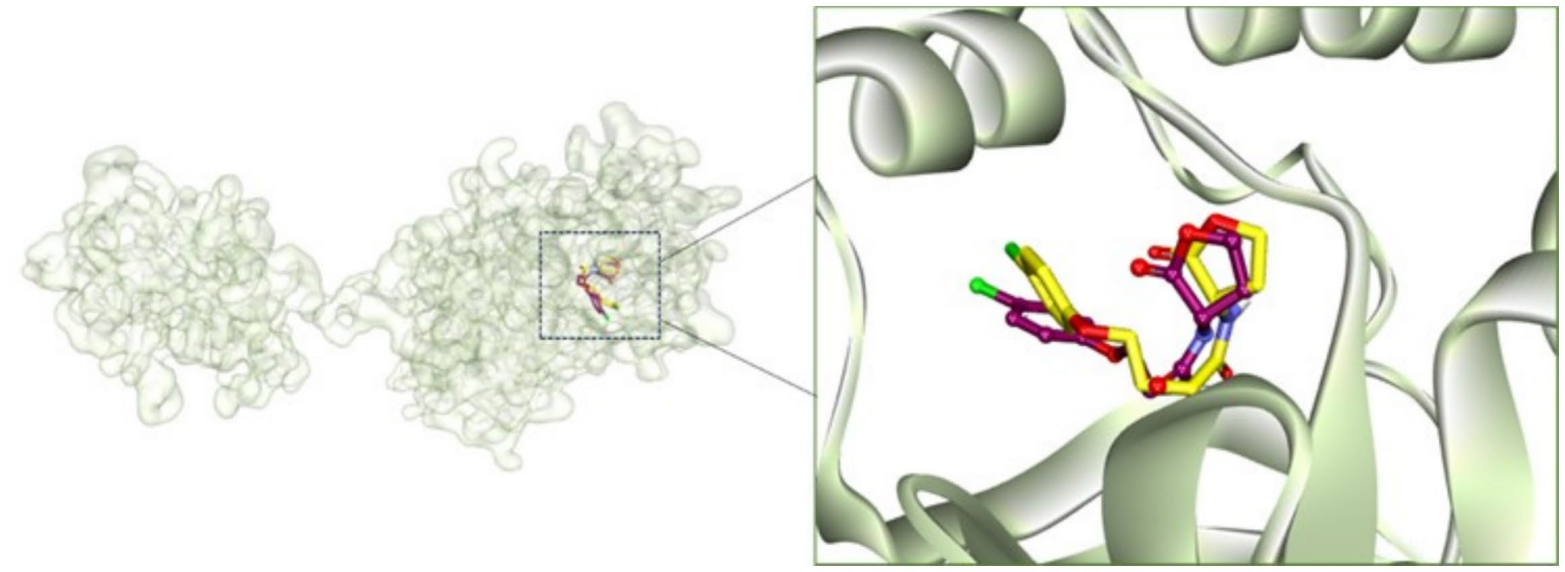
Comparison between crystal structure and redocked conformations of (*S*)-4-(4-chlorophenoxy)-*N*-(2-oxotetrahydrofuran-3-yl)butanamide-CviR complexes. Ball and stick model of ligand in purple colour is the crystal structures conformation and stick model of ligand in yellow colour is the redocked-conformation.

The interaction between certain phytocompounds and proteins or enzymes involved in QS-regulated virulence factors & biofilms was assessed using molecular docking studies to determine the potential mechanism of anti-QS and antibiofilm action of phytocompounds. At first, the docking procedure was validated by extracting (*S*)-4-(4-chlorophenoxy)-*N*-(2-oxotetrahydrofuran-3-yl)butanamide (natural ligand) from receptor-ligand complex followed by redocking. It is evident from Figure 7, that the natural ligand occupied the same position in the active site of CviR (3QP5) as it was present earlier in the crystal structure.

The following QS-related proteins, such as CviR (3QP5), LasA (3IT7), and EsaI (1KZF), as well as biofilm-associated protein, viz. PilY1 (3HX6) were included in the present study.

#### 3.6.1 Interaction of phytocompounds of MKCF with the proteins involved in quorum sensing and biofilms

Molecular docking studies were conducted to better understand the interaction between the identified phytocompounds in MKCF and the proteins or enzymes involved in QS-mediated virulence factors and biofilms. Table 10 lists the binding energies of the phytocompounds of MKCF that show the highest affinity. Among the identified compounds, morellin was found to interact with LasA, CviR, EsaI, and PilY1 most strongly with binding energy of −8.48, −8.29, −9.88, and −8.73 kcal mol^−1^, respectively, compared to other phytocompounds. Similar to morellin, murrayazolinol also showed a good binding affinity with CviR and EsaI with binding energy of −9.07 and −9.17 kcal mol^−1^, respectively.

**Table 10:** Binding energies (kcal mol^−1^) of different ligand-receptor complexes for the phytocompounds of MKCF obtained by molecular docking using Autodock Vina.

Phytocompounds	Proteins involved in quorum sensing and biofilms			
	3IT7 (LasA)	3QP5 (CviR)	1KZF (EsaI)	3HX6 (PilY1)
Murrayazolinol	−7.66	−9.07	−9.17	−7.55
Ismine	−5.46	−7.57	−6.72	−5.81
Morellin	−8.48	−8.29	−9.88	−8.73
Gibberellic acid, methyl ester	−7.35	−8.10	−7.32	−7.11
7,8-Epoxylanostan-11-ol, 3-acetoxy-	−7.14	−9.00	−9.29	−8.56

**Figure 8:**
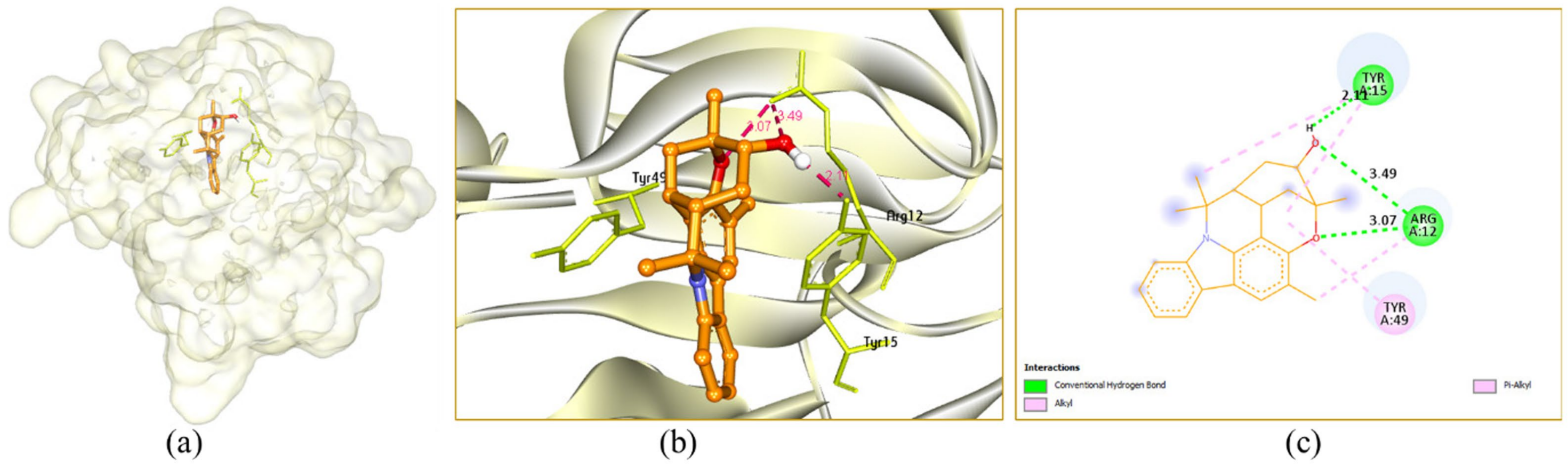
Interaction of murrayazolinol with the receptor 3IT7: **(a)** Surface view expressing the exact location of murrayazolinol in the receptor cavity, **(b)** 3D view showing H-bonding interactions of murrayazolinol with receptor residues in red dashed lines, **(c)** 2D view showing H-bonding interactions (green) with bond distance and other interactions.

In the active site cavity of 3IT7: Murrayazolinol showed three H-bonding interactions with Arg12 (3.07 and 3.49 Å) and Tyr15 (2.11 Å), it showed alkyl and *π*-alkyl hydrophobic interactions with Arg12 (3.97 Å), Tyr15 (4.85 and 5.46 Å) and Tyr49 (4.54 Å) (Figure 8).

**Figure 9:**
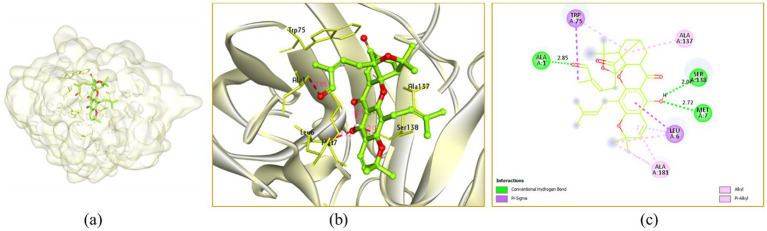
Interaction of morellin with the receptor 3IT7: **(a)** Surface view expressing the exact location of morellin in the receptor cavity, **(b)** 3D view showing H-bonding interactions of morellin with receptor residues in red dashed lines, **(c)** 2D view showing H-bonding interactions (green) with bond distance and other interactions.

Morellin exhibited three hydrogen bonding interactions with Ala1 (2.85 Å), Met7 (2.07 Å) and Ser138 (2.72 Å), it showed alkyl and *π*-alkyl hydrophobic interactions with Leu6 (4.10 and 4.66 Å), Trp75 (5.19 and 5.46 Å), Ala137 (4.40 Å) and Ala181 (3.37, 3.50 and 4.32 Å) (Figure 9).

Gibberellic acid, methyl ester exhibited three H-bonding interactions with Arg12 (3.34 Å), Trp17 (2.81 Å) and Ser50 (2.16 Å), it showed *π*-alkyl hydrophobic interactions with Tyr15 (5.49 Å), Tyr39 (4.02 and 4.95 Å) and Tyr49 (4.33, 4.82 and 5.38 Å) (Supplementary material SI 4).

7,8-Epoxylanostan-11-ol, 3-acetoxy- showed two H-bonding interactions with Ser115 (2.24 Å) and Thr117 (3.33 Å), it showed π-alkyl hydrophobic interaction with Tyr90 (4.26 and 4.90 Å) (Supplementary material SI 5).

**Figure 10:**
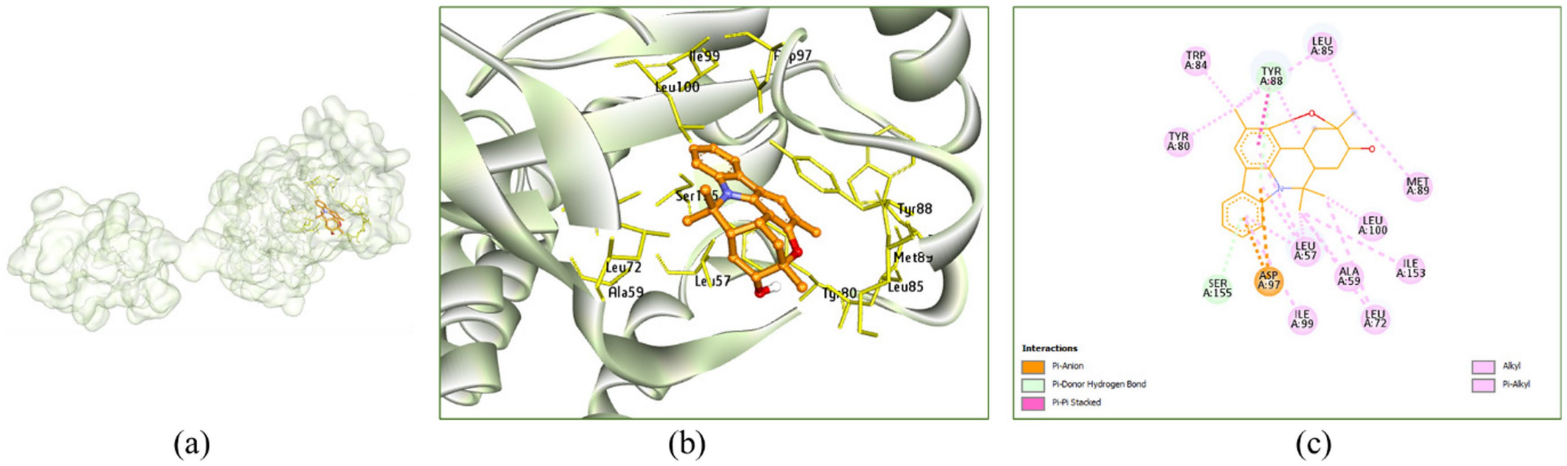
Interaction of murrayazolinol with the receptor 3QP5: **(a)** Surface view expressing the exact location of murrayazolinol in the receptor cavity, **(b)** 3D view showing the nearby residues in the receptor cavity, **(c)** 2D view showing the interactions.

In the active site cavity of 3QP5: Murrayazolinol exhibited no H-bonding interaction however, it showed alkyl and π-alkyl hydrophobic interactions with Leu57 (4.48, 4.91, 5.16 and 5.46 Å), Ala59 (3.91 Å), Leu72 (4.39 and 4.58 Å), Tyr80 (4.46 Å), Trp84 (3.87 Å), Leu85 (4.20 and 4.22 Å), Tyr88 (3.72 and 5.13 Å), Met89 (4.52 Å), Ile99 (5.11 Å), Leu100 (3.49 Å) and Ile153 (4.89 Å) (Figure 10).

**Figure 11:**
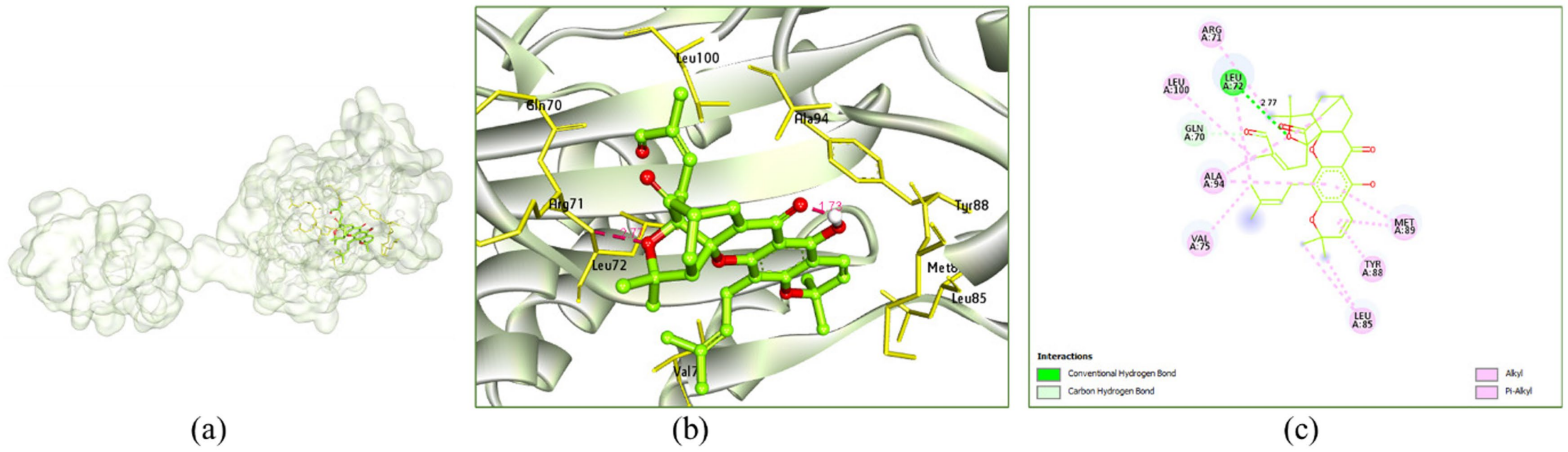
Interaction of morellin with the receptor 3QP5: **(a)** Surface view expressing the exact location of morellin in the receptor cavity, **(b)** 3D view showing H-bonding interactions of morellin with receptor residues in red dashed lines, **(c)** 2D view showing H-bonding interactions (green) with bond distance and other interactions.

Morellin showed H-bonding with Leu72 (2.77 Å), it showed alkyl and *π*-alkyl hydrophobic interactions with Arg71 (4.26 Å), Leu72 (4.61 Å), Val75 (4.45 Å), Leu85 (4.30 and 4.70 Å), Tyr88 (4.99 Å), Met89 (4.54 and 5.39 Å), Ala94 (4.07 and 5.09 Å) and Leu100 (4.64 Å) (Figure 11).

Gibberellic acid, methyl ester showed no H-bonding interaction however, it showed alkyl and *π*-alkyl hydrophobic interactions with Tyr80 (5.45 Å), Trp84 (4.17 Å), Leu85 (4.22 Å), Tyr88 (4.97 Å), Ile99 (4.87 and 5.47 Å), Leu100 (4.51 and 4.73 Å) and Trp111 (5.50 Å) (Supplementary material SI 6).

7,8-Epoxylanostan-11-ol, 3-acetoxy- showed H-bonding interaction with Leu72 (2.93 Å), it showed alkyl and *π*-alkyl hydrophobic interactions with Leu57 (3.65 Å), Leu72 (5.11 Å), Tyr80 (4.06 and 4.65 Å), Trp84 (5.22 Å), Leu85 (4.66 and 4.95 Å), Met89 (4.21, 4.76 and 5.12 Å), Trp111 (5.16 Å) and Met135 (5.09 Å) (Supplementary material SI 7).

In order to investigate the potential mode of action of MKCF phytoconstituents, molecular docking was also carried out using PilY1, a protein associated with biofilms.

**Figure 12:**
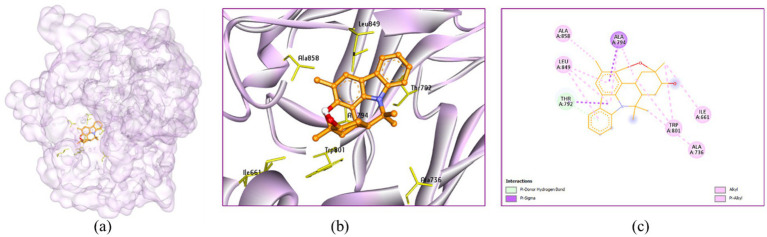
Interaction of murrayazolinol with the receptor 3HX6: **(a)** Surface view expressing the exact location of murrayazolinol in the receptor cavity, **(b)** 3D view showing the nearby residues in the receptor cavity, **(c)** 2D view showing the interactions.

In the active site cavity of 3HX6: Murrayazolinol exhibited no hydrogen bonding interaction however, it showed alkyl and π-alkyl hydrophobic interactions with Ile661 (4.40 Å), Ala736 (4.38 Å), Ala794 (3.99 and 4.68 Å), Trp801 (5.02 and 5.39 Å), Leu849 (5.10, 5.11 and 5.22 Å) and Ala858 (4.14 Å) (Figure 12).

**Figure 13:**
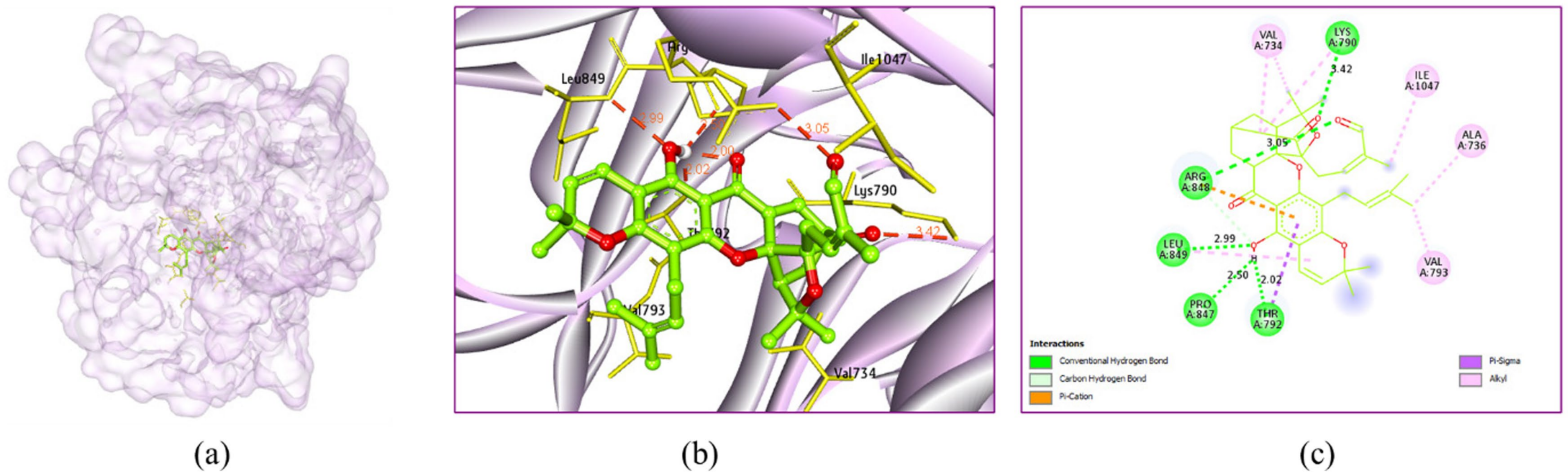
Interaction of morellin with the receptor 3HX6: **(a)** Surface view expressing the exact location of morellin in the receptor cavity, **(b)** 3D view showing H-bonding interactions of morellin with receptor residues in red dashed lines, **(c)** 2D view showing H-bonding interactions (green) with bond distance and other interactions.

Morellin showed five H-bonding interactions with Lys790 (3.42 Å), Thr792 (2.02 Å), Pro847 (2.50 Å), Arg848 (3.05 Å) and Leu849 (2.99 Å). It showed alkyl hydrophobic interactions with Val734 (3.93 and 4.68 Å), Ala736 (3.16 Å), Lys790 (4.74 Å), Val793 (4.89 Å), Leu849 (5.38 Å) and Ile1047 (4.39 Å) (Figure 13).

Gibberellic acid, methyl ester showed two H-bonding interactions with Arg795 (2.88 Å) and Gly856 (1.86 Å), it exhibited alkyl and π-alkyl hydrophobic interactions with Leu657 (4.00 and 4.60 Å), Ala658 (4.28 Å), Ile661 (4.37 Å), Ala794 (5.04 Å) and Trp801 (4.78 Å) (Supplementary material SI 8).

7,8-Epoxylanostan-11-ol, 3-acetoxy- showed H-bonding interaction with Leu927 (2.84 Å), it showed alkyl and π-alkyl hydrophobic interactions with Tyr653 (4.09 and 5.41 Å), Ala794 (4.97 Å), Trp801 (5.42 Å), Arg848 (5.42 Å), Leu849 (4.20 Å), Ala850 (5.31 Å), Ala858 (3.86 Å) and Leu927 (4.10 Å) (Supplementary material SI 9).

## 4 Discussion

An imbalance between pro-oxidants and antioxidant species is known as oxidative stress, and it causes damage to molecules and cells. Research in the last few decades has provided evidence that natural substances from plants have great potential to mitigate oxidative stress and enhance protective and immunological function (Pisoschi and Pop, 2015; Ricordi et al., 2015; Tan et al., 2018). It has been recognized that exogenous reducing agents like polyphenols, vitamin E, carotenoids, and vitamin C play an essential role to completely neutralize excess reactive species derived from oxygen produced during regular cellular metabolism by using molecular oxygen, such as during processes like mitochondrial respiration, which metabolizes 85% of inhaled oxygen (Bouayed and Bohn, 2010).

The most active fraction of *M. koenigii* was found to be chloroform, with an IC_50_ value of 28.59 μg/mL, while the lowest activity was expressed in the hexane fraction with an IC_50_ value of 98.43 μg/mL in the DPPH inhibition assay. The ferric-reducing activity was recorded highest for chloroform, followed by the ethyl acetate fraction. The hexane fraction showed the least reducing power. The findings corroborate earlier finding where the chloroform fraction showed DPPH radical scavenging activity with an IC_50_ value of 24.62 μg/mL (Dahlia et al., 2017). Bacterial quorum sensing regulates virulence factors in *P. aeruginosa* and other Gram-negative bacteria. Targeting QS can attenuate the virulence of pathogenic bacteria by killing it. Therefore, QS is considered a promising anti-infective drug target (Papenfort and Bassler, 2016). The current research priority is searching for a novel, safe and effective anti-QS agent from natural products. In this paper, the most antioxidant-active fraction of *M. koenigii*, i.e., MKCF, was tested at sub-MIC values against test strains. Interestingly, it should be noted that at the highest tested sub-MIC of 1,000 μg/mL, maximum inhibition of virulence factors was observed. The findings revealed a 70.73% reduction of the violacein pigment in the presence of MKCF. It was discovered that following MKCF treatment, the amount of pyocyanin was considerably reduced in a concentration-dependent way. The maximum measured concentration of 1,000 μg/mL MKCF resulted in 64.75, 54.39, 56.66, 55.88, and 56.28% inhibition of pyocyanin, pyoverdin, protease activity, rhamnolipid, and swarming motility, respectively. Additionally, at 1000 μg/mL MKCF, there was 62.26, 68.75, and 76.03% inhibition of prodigiosin production, protease activity, and swarming motility of *S. marcescens*. The results of violacein and pyocyanin inhibition in the presence of MKCF support earlier findings where the essential oil of *M. koenigii* have been reported to inhibit the swimming motility and production of pyocyanin in *P. aeruginosa* PAO1 (Bai Aswathanarayan and Vittal, 2014).

MKCF exhibited a concentration-dependent inhibitory response on the formation of bacterial biofilm at respective sub-MIC values. The process of biofilm formation is extremely regulated and organized, and it is intimately linked to bacterial cellular communication, specifically QS (Qin et al., 2014). The assessment of antibiofilm activity against *P. aeruginosa* PAO1 showed that MKCF exhibited maximum biofilm inhibition by 66.07% at 1000 μg/mL. Similarly, 1,000 μg/mL MKCF reduced the biofilms of *C. violaceum* 12,472 by 76.62%, compared to the untreated control. A similar concentration-dependent inhibitory response was also recorded against *S. marcescens*. A significant level of biofilm inhibition against different test pathogens demonstrated the broad-spectrum antibiofilm activity of MKCF. Our findings are in agreement with reports published on other bioactive plant extracts against two or more bacterial pathogens (Mehrishi et al., 2020; Muruzović et al., 2016; Muthusamy and Girija, 2020; Nostro et al., 2016). Such bioactive plant extracts could be developed into effective formulations to be exploited as standardized extracts/herbal formulations or in combinational therapy. The presence of polyphenolic components in the extract or fractions is one of the most frequent causes of biofilm inhibition by plant extract (Maisuria et al., 2015; Zhang et al., 2014). Recent studies on *M. koenigii* have also highlighted its neuroprotective, cardioprotective, and immunomodulatory effects. While *in vitro* studies provide promising insights, further investigations through *in vivo* and clinical models are essential to validate the safety, bioavailability, and efficacy of its phytoconstituents. Previously, acute toxicity study of *M. koenigii* was carried out against Swiss albino mice, which showed no mortality at the highest dose level and did not possess any toxic effect and are safe till the dose level of 9,000 mg/kg (Saini and Reddy, 2013).

TLC-based detection of the major groups of phytocompounds showed the presence of terpenoids, flavonoids, and alkaloids in the most bioactive MKCF. GC/MS and LC-qTOF/MS analyses of the most active fractions of *M. koenigii* demonstrated the presence of various phytocompounds belonging to major groups such as alkaloids, polyphenols, tannins, and terpenoids. The observed bioactivity of *M. koenigii* chloroform fraction is indeed due to the synergistic effects of multiple compounds rather than a single compound. Previous studies have shown that these compounds work together to exhibit significant antioxidant, antimicrobial, and anti-inflammatory effects, indicating that the combination of these phytochemicals enhances their overall bioactivity (Joshi and Bisht, 2025). In MKCF, some major compounds detected by GC/MS analysis with high percent peak area are Gibberellic acid, methyl ester (7.16%), 7,8-Epoxylanostan-11-ol, 3-acetoxy- (5.74%), 1′,1’-Dicarboethoxy-1β,2β-dihydro-3’H-cycloprop (Maisuria et al., 2016; Rutherford and Bassler, 2012) cholesta-1,4,6-trien-3-one (3.73%), 3,9- Epoxypregn-16-ene-14,20-diol,7,11,18-triacetoxy-3-methoxy- (2.32%) respectively. Some phytocompounds identified by LC-qTOF/MS analysis were murrayazolinol, ismine, morellin, bismahanine, phytosphingosine. In a previous study, four C23-carbazole alkaloids, viz., mahanimbine, murrayamine-J, murrayazolinol, and bicyclomahanimbine, were isolated and identified as the chemical components of *M. koenigii* (Tan et al., 2014). Bismahanine was previously isolated from the leaves of *M. koenigii* (Chatterjee et al., 2023).

There are different approaches to assess the mechanism of action of phytocompounds at the molecular level, such as *in silico* studies and differential gene expression. Molecular docking experiments were carried out utilizing AutoDock Vina to gain a deeper understanding of the interaction mechanism of the phytocompounds identified in MKCF to the proteins or enzymes involved in biofilm formation and quorum-sensing mediated virulence factor. Previously, the docking score of 3ZH5 protein E with murrayazolinol was −6.9 (Ganesh, 2022).

## 5 Conclusion

The study’s findings indicated that the most active fraction of *M. koenigii*, i.e., MKCF, displayed anti-infective efficacy *in vitro* against test pathogens. A concentration-dependent suppression of QS-controlled virulence factors and biofilms highlights its broad-spectrum activity The major phytocompounds identified by GC/MS and LC-qTOF/MS analysis demonstrated a moderate affinity for binding to QS and biofilm-associated proteins of *P. aeruginosa* PAO1 and *C. violaceum* 12,472, which further strengthens our findings. While these preliminary findings are encouraging, further validation through *in vivo* models and mechanistic studies is essential to assess therapeutic potential.

## Data Availability

The raw data supporting the conclusions of this article will be made available by the authors, without undue reservation.

## Footnotes

1
https://pubchem.ncbi.nlm.nih.gov/compound/1794427

2
https://www.rcsb.org/pdb/home/home.do

3
http://sts.bioe.uic.edu/castp/index.html?3it7
