# Supplementary material for: Anti-infective potential, chemical profile, and molecular docking investigation on antioxidant-rich fraction of Murraya koenigii against Gram-negative pathogenic bacteria
Source: Front Microbiol. 2026 Feb 4;17:1739591. doi: 10.3389/fmicb.2026.1739591 (PMC12913480; doi:10.3389/fmicb.2026.1739591)
Supplement: Supplementary file 1 [file Table_1.DOCX]

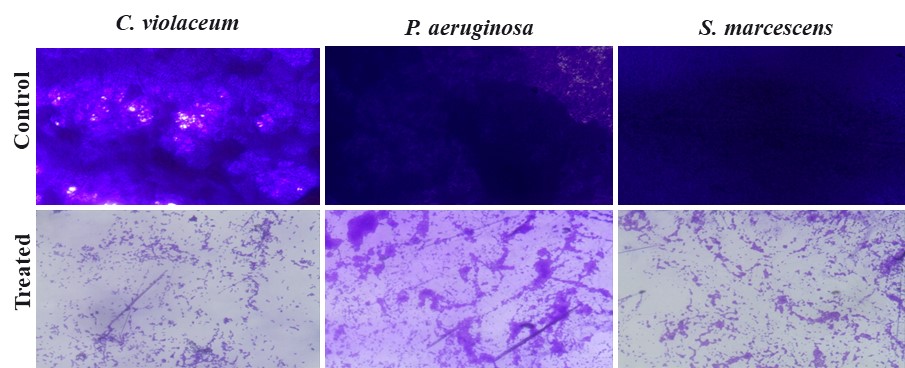


**SI 1.** Light microscopic images of *C. violaceum*, *P. aeruginosa,* and *S. marcescens* biofilm in the absence and presence of sub-MIC (1000 µg/ml) of MKCF.

**SI 2.** Gas chromatography mass spectrometry (GC/MS) chromatogram of MKCF.


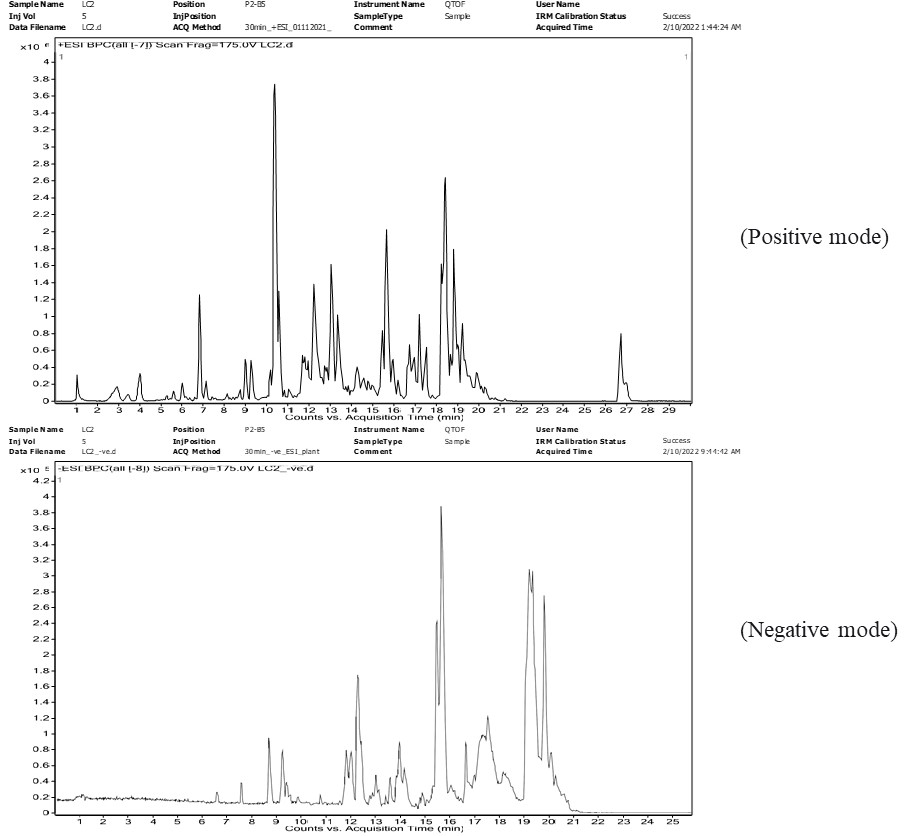


**SI 3.** Liquid Chromatography mass spectrometry (LC/MS) chromatogram of MKCF.


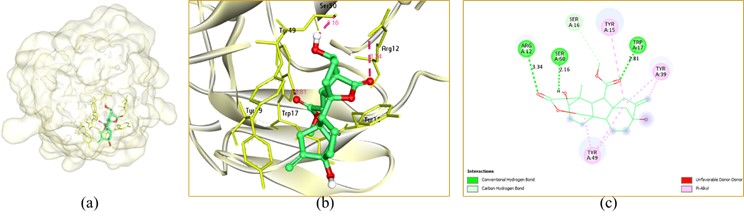


**SI 4.** Interaction of gibberellic acid, methyl ester with the receptor 3IT7: (a) surface view expressing the exact location of gibberellic acid, methyl ester in the receptor cavity, (b) 3D view showing H-bonding interactions of gibberellic acid, methyl ester with receptor residues in red dashed lines, (c) 2D view showing H-bonding interactions (green) with bond distance and other interactions.


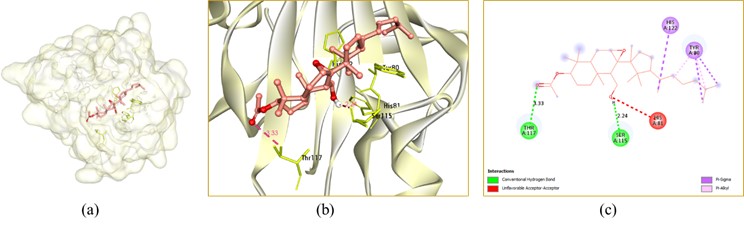


**SI 5.** Interaction of 7,8-Epoxylanostan-11-ol, 3-acetoxy- with the receptor 3IT7: (a) surface view expressing the exact location of 7,8-Epoxylanostan-11-ol, 3-acetoxy- in the receptor cavity, (b) 3D view showing H-bonding interactions of 7,8-Epoxylanostan-11-ol, 3-acetoxy- with receptor residues in red dashed lines, (c) 2D view showing H-bonding interactions (green) with bond distance and other interactions.


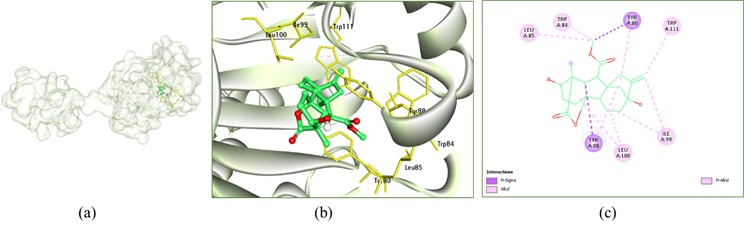


**SI 6.** Interaction of gibberellic acid, methyl ester with the receptor 3QP5: (a) surface view expressing the exact location of gibberellic acid, methyl ester in the receptor cavity, (b) 3D view showing the nearby residues in the receptor cavity, (c) 2D view showing the interactions.

**
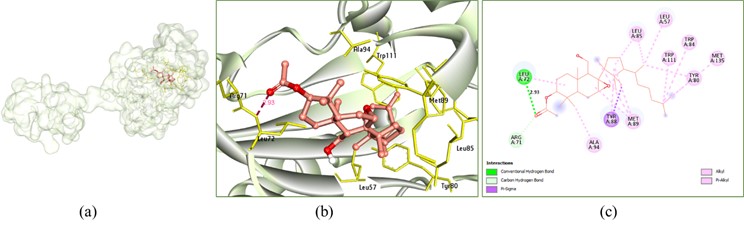
**

**SI 7.** Interaction of 7,8-Epoxylanostan-11-ol, 3-acetoxy- with the receptor 3QP5: (a) surface view expressing the exact location of 7,8-Epoxylanostan-11-ol, 3-acetoxy- in the receptor cavity, (b) 3D view showing H-bonding interactions of 7,8-Epoxylanostan-11-ol, 3-acetoxy- with receptor residues in red dashed lines, (c) 2D view showing H-bonding interactions (green) with bond distance and other interactions.


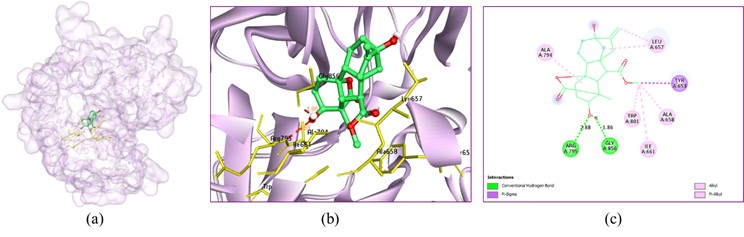


**SI 8.** Interaction of gibberellic acid, methyl ester with the receptor 3HX6: (a) surface view expressing the exact location of gibberellic acid, methyl ester in the receptor cavity, (b) 3D view showing H-bonding interactions of gibberellic acid, methyl ester with receptor residues in red dashed lines, (c) 2D view showing H-bonding interactions (green) with bond distance and other interactions.


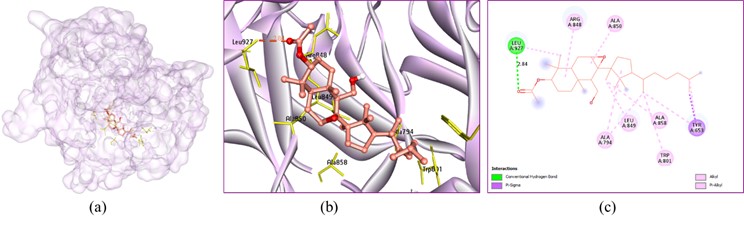


**SI 9.** Interaction of 7,8-Epoxylanostan-11-ol, 3-acetoxy- with the receptor 3HX6: (a) surface view expressing the exact location of 7,8-Epoxylanostan-11-ol, 3-acetoxy- in the receptor cavity, (b) 3D view showing H-bonding interactions of 7,8-Epoxylanostan-11-ol, 3-acetoxy- with receptor residues in red dashed lines, (c) 2D view showing H-bonding interactions (green) with bond distance and other interactions.

SI 10. TLC-based detection of phytochemical classes.

| **Phytochemical class** | **Reagent used** | **Observation** | **Inference** |
| --- | --- | --- | --- |
| Flavonoids | Natural product reagents | Orange, orange-yellow or yellow-green fluorescent bands | Presence of flavonoids |
| Terpenoids | Vanillin-sulfuric acid | Red or purple spots/bands | Presence of terpenoids |
| Alkaloids | Dragendorff’s reagent | Orange-brown bands | Presence of alkaloids |
